# The Pschorr Reaction: Recent Advances and Application in Heterocyclic Synthesis

**DOI:** 10.3390/molecules31091398

**Published:** 2026-04-23

**Authors:** Rodrigo Abonia, Daniel Insuasty, Juan-Carlos Castillo, Kenneth K. Laali

**Affiliations:** 1 Research Group of Heterocyclic Compounds, Department of Chemistry, Universidad del Valle, Cali A. A. 25360, Colombia; rodrigo.abonia@correounivalle.edu.co; 2 Grupo de Investigación en Química y Biología, Departamento de Química y Biología, Universidad del Norte, Km 5 vía Puerto Colombia 1569, Barranquilla Atlántico 081007, Colombia; insuastyd@uninorte.edu.co; 3 Escuela de Ciencias Químicas, Universidad Pedagógica y Tecnológica de Colombia, Avenida Central del Norte 39-115, Tunja 150003, Colombia; juan.castillo06@uptc.edu.co; 4 Department of Chemistry and Biochemistry, University of North Florida, 1 UNF Drive, Jacksonville, FL 32224, USA

**Keywords:** Pschorr reaction, diazonium salts, intramolecular cyclizations, metal-catalyzed or thermal reactions, synthesis of heterocycles

## Abstract

The Pschorr reaction is a radical-mediated intramolecular cyclization involving diazonium salts, affording five-, six- and seven-membered fused polycyclic and heterocyclic rings, discovered by R. Pschorr in the late nineteenth century. Over the years, this classic reaction has played an important role in ring-forming reactions. In 2009 we reviewed the progress in the field. The intervening years have witnessed major advances in the application of Pschorr reaction that are mediated by various metals, by photocatalysis, and by ionic liquids, leading to the development of new and improved methods for the synthesis of diverse bioactive heterocycles. The notable progress in the field since our 2009 review provided the impetus to summarize, discuss, and put these advances in perspective.

## 1. Introduction

The Pschorr reaction [1], along with the Sandmeyer [2], Gomberg–Gatterman [3], Balz–Schiemann [4], and Meerwein [5] reactions, forms a classical dynamic quintet for the introduction of aryl groups, by forming aryl radicals or aryl cations via homolytic or heterolytic dediazoniation, starting from aryldiazonium salts. Collectively, these reactions have had a tremendous impact on synthetic/preparative organic chemistry for the preparation of high-value chemicals, as well as the synthesis of polycyclic aromatic hydrocarbons (PAHs) and hetero-PAHs. As a radical-mediated intramolecular cyclization process, the Pschorr reaction stands out as a classic method for the synthesis of five-, six- and seven-membered fused polycylic and heterocyclic rings. The common link in all of these “name reactions” is the formation of aryl electrophiles from aryldiazonium salts.

The last two decades have witnessed a revival of interest in diazonium chemistry [6,7,8,9,10]. In connection to our long-standing interest in the mechanistic and synthetic aspects of dediazoniation and diazonium ion chemistry [11,12,13,14,15,16,17], and in view of the recent advances that have led to the development of newer methods for the synthesis of diverse bioactive heterocycles, herein we summarize and discuss the progress in this area during the past 15 years.

## 2. Metal-Redox-Catalyzed Pschorr Reaction

### 2.1. Palladium-Catalyzed Pschorr Reaction

#### Synthesis of Benzochromenes

**Scheme 1:**
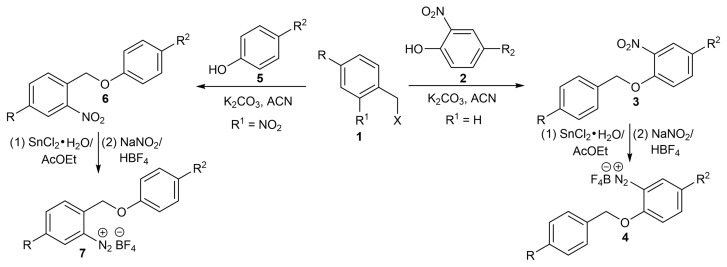
Synthetic approaches for the synthesis of the key diazonium salts **4** and **7**.

The benzochromene moiety is present in diverse natural products and bioactive molecules [18]. A Pd-catalyzed Pschorr reaction provided facile access to 6*H*-benzo[*c*]chromene derivatives **8** and **9** (Table 1 and Table 2) by intramolecular cyclization of *o*-diazonium tetrafluoroborates **4** and **7**, respectively [19]. The key diazonium precursors **4** and **7** were obtained from a two-step sequence starting from benzyl halides **1**, via etherification, reduction, and diazotization according to Scheme 1.

Subsequent treatment of the diazonium salts **4** with Pd(OAc)_2_ and K_2_CO_3_ under reflux in ACN afforded the corresponding 6*H*-benzo[*c*]chromenes **8** (Table 1).

Optimization studies showed that Pd(OAc)_2_ was superior to Pd(PPh_3_)_4_ and Pd(ACN)_2_Cl_2_, as these catalysts gave lower yields. Screening of solvents showed that ACN was an optimal solvent, as DMF, EtOH and acetone gave lower yields, and for the base employed, K_2_CO_3_ proved superior to Na_2_CO_3_, Cs_2_CO_3_, TEA or to no base. Treatment of the diazonium salts **7** under the established conditions afforded the respective 6*H*-benzo[*c*]chromenes **9** (Table 2).

The method exhibited wide functional group tolerance (i.e., Cl, Br, OMe, *t*Bu, etc.) and substitution patterns, starting from precursors **1**, **2** and **5**.

**Scheme 2:**
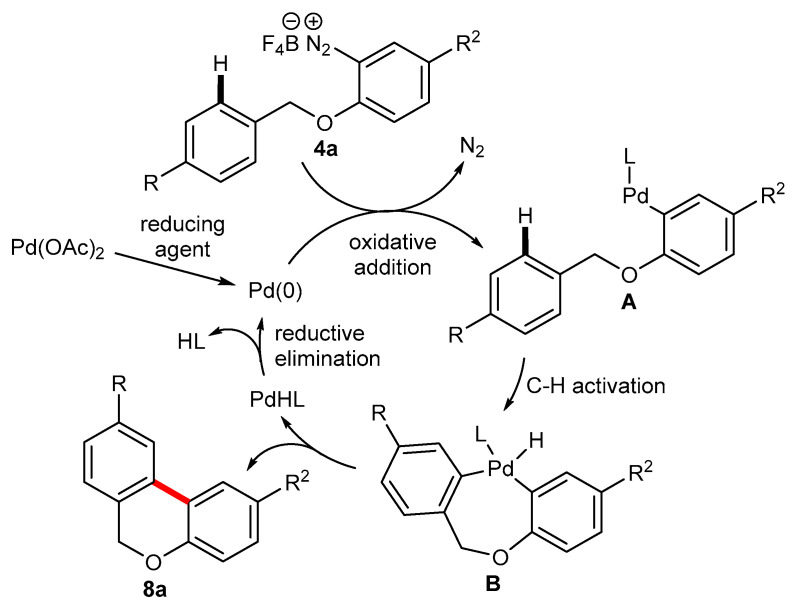
Plausible mechanism for the Pd-mediated intramolecular cyclization of the diazonium salt **4a** via Pschorr reaction.

The suggested mechanism for the intramolecular cyclization of the diazonium salt **4a** (R,R^2^ = H) (Scheme 2) involves reduction of Pd(II) to Pd(0), formation of species **A** by oxidative addition with release of N_2_, intramolecular C–H insertion of Pd to form the tricyclic species **B**, and reductive elimination to give **8a**.

### 2.2. Copper-Catalyzed Pschorr Reaction

#### 2.2.1. Total Synthesis of the 4,5-Didehydroguadiscine **18a**

**Scheme 3:**
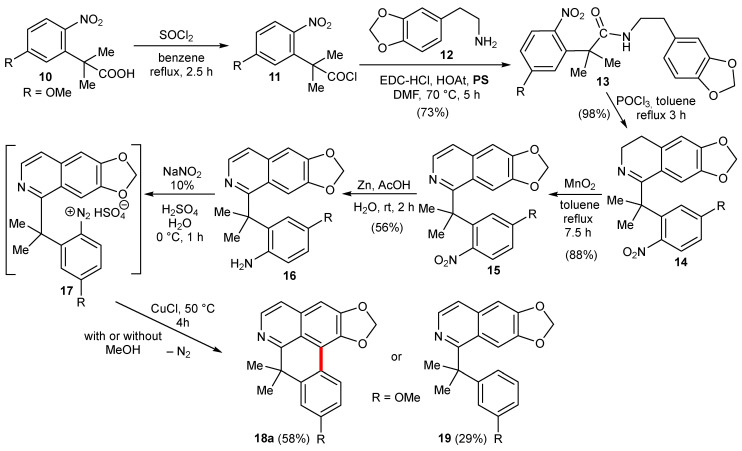
Total synthesis of the 4,5-didehydroguadiscine **18a** from the aniline precursor **16**, via a Cu-catalyzed Pschorr cyclization.

This compound belongs to a diverse family of isoquinoline alkaloids, an aporphinoid with potent melanogenesis-inhibitory activity. The synthetic sequence of **18a** incorporated a Pschorr cyclization on a benzylisoquinoline intermediate (Scheme 3) [20]. The nitrophenylpropanoic acid **10** was converted to acyl chloride **11** by treatment with SOCl_2_ which was then coupled with piperonylamine **12** using Carpino’s peptide coupling method [21], to form the amide intermediate **13**. An intramolecular POCl_3_-mediated cyclization of **13** generated the isoquinoline nucleus **14**, which upon dehydrogenation with MnO_2_ furnished the aromatic isoquinoline **15**. Selective reduction of the nitro group using zinc in acidic medium produced the aniline **16**, which was treated with NaNO_2_/H_2_SO_4_, affording the corresponding diazonium salt **17**. Finally, the Pschorr reaction was accomplished by treatment of the diazonium salt **17** with CuCl under methanol-free conditions, promoting the intramolecular radical cyclization, leading to the formation of the expected tetracyclic 4,5-didehydroguadiscine framework **18a** (R = OMe) (Scheme 3).

In the presence of MeOH, a competing single-electron reduction pathway of the diazonium intermediate **17** occurred, generating the side-product **19**. This finding highlights the influence of the solvent on the regioselectivity of the process. Pharmacological assays indicated that compound **18a** has significant melanogenesis inhibition (IC_50_ = 4.7 μM), making it approximately 40 times more potent than arbutin (IC_50_ = 174 μM), the reference drug, positioning compound **18a** as the most active natural inhibitor within this family of compounds.

#### 2.2.2. Synthesis of Diversely Substituted 4,5-Didehydroguadiscines **18**

**Scheme 4:**
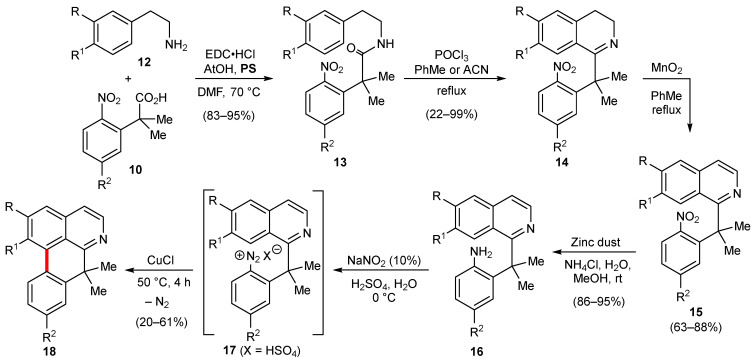
Synthesis of 4,5-didehydroguadiscines **18** via a CuCl-catalyzed Pschorr reaction from the aniline precursor **16**.

As mentioned before, the 4,5-didehydroguadiscine **18a** (R,R^1^ = OCH_2_O; R^2^ = OMe) (Scheme 4) is a naturally occurring alkaloid with remarkable melanogenesis-inhibitory activity. It features an aporphine scaffold with an aromatized B-ring, a structural motif that has received limited attention in structure–activity relationship (SAR) studies [22,23]. In this context, a further approach for the synthesis of a series of 4,5-didehydroguadiscine derivatives **18** was reported (Scheme 4) [24]. The synthetic route involves coupling of nitrocarboxylic acids **10** and phenethylamines **12** in the presence of EDC·HCl, AtOH, and a proton sponge (**PS**) to afford amides **13**. Subsequently, the Bischler–Napieralski cyclization of amides **13** in toluene afforded the corresponding dihydroisoquinoline derivatives **14**. For amides **13** lacking activating substituents, derivatives **14** could still be obtained by using ACN as solvent albeit in lower yields. The dihydroisoquinolines **14** were efficiently converted into the corresponding nitro-isoquinolines **15** by a MnO_2_-mediated oxidative dehydrogenation reaction. The resulting nitro-substituted intermediates **15** were then reduced by treatment with zinc dust in the presence of NH_4_Cl to furnish aniline derivatives **16**. Diazotization of **16** gave the corresponding diazonium salts **17**, which in the presence of CuCl as catalyst underwent an intramolecular Pschorr reaction to afford 4,5-didehydroguadiscines **18** (Scheme 4 and Table 3).

##### Bioactivity Study

Compounds **18** were tested for melanogenesis-inhibitory effects against theophylline-stimulated B16 melanoma 4A5 cells in the concentration range of 1–30 μM, using the MTT assay. Results showed that 4,5-didehydroguadiscine derivatives **18b–f** are highly potent, with IC_50_ values ranging from 3.1 to 7.5 µM, with some of them showing similar or better activity compared to the naturally occurring 4,5-didehydroguadiscine **18a** (IC_50_ = 4.9 μM, Table 3). Interestingly, the unsubstituted derivative **18e** (R = R^1^ = R^2^ = H) exhibited the highest potency, with IC_50_ = 3.1 µM, around 56-fold enhancement in activity relative to arbutin (IC_50_ = 174 μM) [25], indicating that the absence of the *O*-substituents in **18** did not significantly affect the melanogenesis-inhibitory activity (Table 3).

#### 2.2.3. Synthesis of the Methyl 6H-Benzo[e]naphtho[2,3-c][1,2]thiazine-10-Carboxylate 5,5-Dioxide **26**

**Scheme 5:**
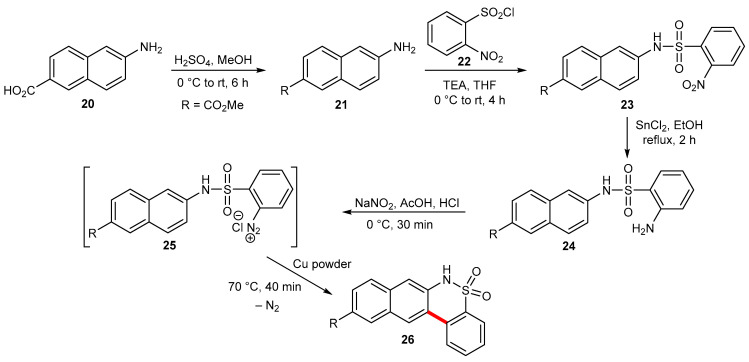
Synthesis of the methyl 6*H*-benzo[*e*]naphtho[2,3-*c*][1,2]thiazine-10-carboxylate 5,5-dioxide **26** via a copper-catalyzed Pschorr reaction from the key aniline precursor **24**.

Cyclin-dependent kinase 4 (Cdk4) inhibition is a promising therapeutic strategy for mitigating neurodegeneration in Alzheimer’s disease (AD) [26]. The ester moiety of compound **26** was employed as a pivotal building block for the synthesis of Cdk4 inhibitors with potential neuroprotective activity (Scheme 5). The synthetic route began with 6-aminonaphthalene-2-carboxylic acid **20**, which underwent Fischer esterification with 10% aqueous H_2_SO_4_ in MeOH, affording methyl ester **21**. Subsequently, compound **21** was sulfonylated with 2-nitrobenzenesulfonyl chloride **22** in the presence of TEA in THF, yielding the *N*-sulfonyl derivative **23**. Reduction of the nitro group of **23** with SnCl_2_ in refluxing EtOH furnished the aniline derivative **24**. Diazotization of **24** with sodium nitrite in a AcOH:HCl (3:1, *v*/*v*) mixture generated the diazonium salt **25**, which underwent a copper-catalyzed Pschorr cyclization to yield the target dioxide **26** [26].

### 2.3. Gold-Catalyzed Pschorr Reaction

The fluorenone framework is widely present in bioactive molecules [27] and also serves as a building block in materials applications [28]. The classical approaches to fluorenone synthesis typically rely on Friedel–Crafts acylation or oxidation of fluorene derivatives. These methods employ strong acids, oxidants, or elevated temperatures [29,30]. An alternative strategy for the synthesis of structurally valuable fluorenones **31** employed aryldiazonium salts as electrophilic partners in an Au(I)-catalyzed oxidation/C–H activation/cyclization cascade [31]. In this process, the key aniline precursors **29** were prepared through a two-step sequence involving an initial palladium-catalyzed oxidative C–H coupling between acetanilides **27** and aromatic aldehydes **28**, followed by hydrolysis of the acetamide (COMe) protecting group [32]. Subsequent diazotization of anilines **29** in the presence of *t*BuONO/HPF_6_ afforded the aryldiazonium salts **30**, which were isolated and stored in the dark. After optimization of the reaction conditions, the Pschorr cyclization was accomplished by subjecting the diazonium salts **30** to the Me_2_SAuCl/terpy/ascorbic acid catalytic system, to form the desired fluorenones **31** (Table 4).

**Figure 1:**
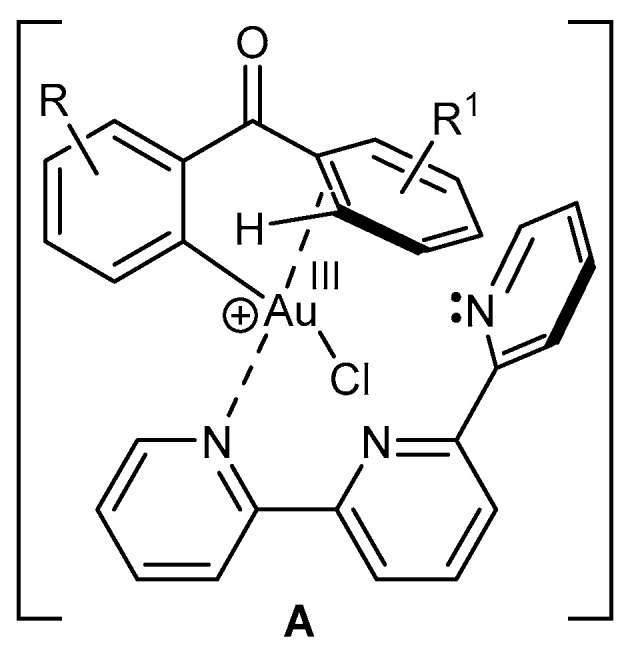
Structure of the Au(III) arene π-complex **A** proposed as the key species in the formation of the fluorenones **31** via a S_E_Ar pathway.

Substituent effect study showed that electron-donating groups on either one of the aryl rings of the aryldiazonium **30** significantly enhanced the efficiency of the reaction, whereas electron-withdrawing substituents markedly suppressed their reactivity (Table 4). The observed trend is consistent with an electrophilic aromatic substitution (S_E_Ar) mechanistic sequence, involving a C–H auration/activation step preceded by an η^2^-coordination with terpy ligand, in which the key Au(III) arene π-complex **A** (Figure 1) should be formed after an oxidation process [33].

## 3. Metal-Free-Catalyzed Pschorr Reaction

### 3.1. Brønsted Acid-Catalyzed Pschorr Reaction

#### 3.1.1. Synthesis of Multicaulin

**Scheme 6:**
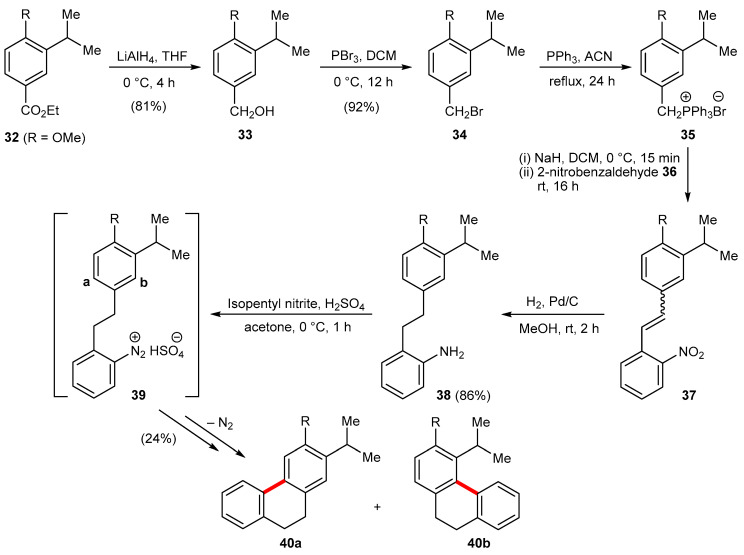
Synthesis of the phenanthrene derivatives **40a**/**40b** via a H_2_SO_4_-catalyzed Pschorr cyclization of the diazonium salt **39**.

Multicaulin is a naturally occurring diterpenoid characterized by a phenanthrene core bearing a methoxy substituent at C–6, methyl groups at C–1 and C–2, and an isopropyl group at C–7, exhibiting notable antitubercular activity [34]. Its successful total synthesis has enabled detailed structure–activity relationship studies and facilitated the preparation of structurally related analogs, including compounds **40a** and **40b** (Scheme 6) [34]. The synthetic sequence commenced with reduction of ester **32** using LiAlH_4_ in THF to furnish alcohol **33** which was converted to benzyl bromide **34** by an Appel-type bromination with PBr_3_ in DCM. Treatment of **34** with PPh_3_ in refluxing ACN generated the phosphonium salt **35**, which was employed directly in the next step without purification. A Wittig olefination between phosphonium salt **35** and 2-nitrobenzaldehyde **36** afforded an inseparable mixture of (*E*/*Z*)-nitrostilbene **37**, which was subsequently hydrogenated over Pd/C in MeOH to deliver aniline derivative **38** in an overall 86% yield across three steps. Diazotization of aniline **38** using isopentyl nitrite in the presence of sulfuric acid generated the corresponding diazonium salt **39**, which underwent a Pschorr reaction to afford an inseparable mixture of products **40a**/**40b** in 3:1 regioisomeric ratio. This outcome reflects the operation of two competing intramolecular aryl–aryl bond-forming pathways occurring at positions “**a**” and “**b**” from the diazonium intermediate **39** (Scheme 6).

##### Bioactivity Study

The inseparable mixture of compounds **40a**/**40b** was subsequently evaluated against *Mycobacterium smegmatis*, *Mycobacterium bovis*, *Mycobacterium szulgai*, *Mycobacterium gastri*, *Mycobacterium simiae*, and *Mycobacterium tuberculosis* H37Ra, using streptomycin as the reference control (Table 5) [34]. Although compounds **40** displayed no significant antimycobacterial activity, with MIC values ranging from 991.50 to >991.50 µM, markedly lower than those of streptomycin (MIC = 0.17–2.68 µM), their structures proved to be valuable synthetic intermediates used as key precursors for the preparation of multicaulin- and miltirone-like compounds, which exhibited remarkable antimycobacterial activity.

### 3.2. Ionic Liquid (IL)-Catalyzed Pschorr Reaction

The Pschorr reaction of arenediazonium salts **30** was investigated in [BMIM][TfO] and [BMIM][Tf_2_N], with particular focus on substituent effects on product distribution (Table 6 and Table 7) [35]. Salts **30** were obtained by reacting their corresponding keto-anilines **29** with sodium nitrite in aq HBF_4_. The ILs were prepared starting from [BMIM][Cl] by metathesis with LiOTf and LiNTf_2_ [36,37,38]. Heating solutions of **30** in [BMIM][TfO] yielded mixtures of 9-fluorenone products **31** and substitution products **41** as determined by ^1^H NMR (Table 6).

Analogously, mixtures of products **31** and substitution products **42** and **43** were obtained when diazonium salts **30** were heated in [BMIM][Tf_2_N] (Table 7). In this case, the Pschorr products **31** were formed more selectively than in [BMIM][TfO], indicating a more facile electron transfer with [Tf_2_N]. Preferential formation of **42** over **43** reflects the dominance of *O*-attack over *N*-attack. Additionally, the nature of the substituents (R = H, Me, MeO, Cl) had no significant influence on product distribution.

**Scheme 7:**
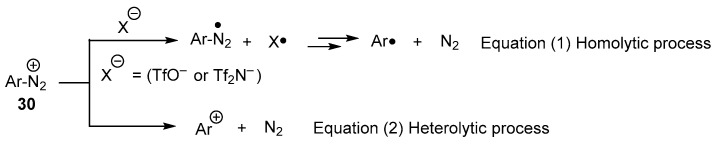
Proposed mechanistic sequence for homolytic and heterolytic dediazoniation of ArN_2_^⊕^ ions from salts **30** and analogs.

The observed product distributions reflect competing homolytic and heterolytic dediazoniations. Aryl radicals Ar• are typically involved in intramolecular cyclizations (Scheme 7, Equation (1)), affording fused systems like **31** via Pschorr reaction, while aryl cations Ar^+^ typically undergo substitution by counterion attack (Scheme 7, Equation (2)), affording substituted products like **41**–**43**. As mentioned earlier, formation of products **41**–**43** reflects the ambident nucleophilic character of [OTf] and [NTf_2_] counterions [16].

DFT computations were performed to estimate the influence of the IL on homolytic versus heterolytic processes with aryldiazonium salts **30** (Scheme 7) and the results suggested that the homolytic process is induced more readily in [BMIM][Tf_2_N] than in [BMIM][TfO].

### 3.3. TEMPO-Catalyzed Pschorr Reaction

**Scheme 8:**
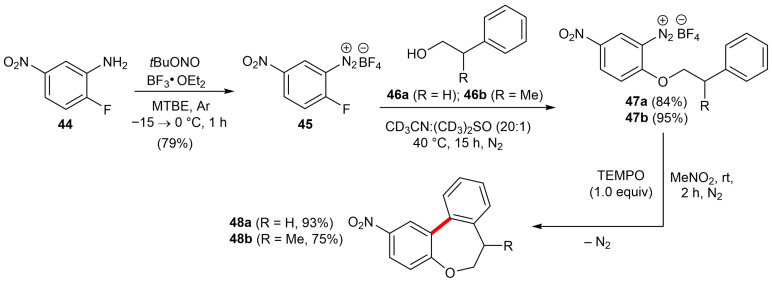
Synthesis of tricyclic dibenzoxepanes **48** via a TEMPO-promoted Pschorr reaction from functionalized aryl–alkyl diazonium ethers **47**.

The 2-fluoro-5-nitrophenyldiazonium tetrafluoroborate **45** has emerged as an efficient Sanger-type reagent for the direct functionalization of aliphatic alcohols like **46** (Scheme 8) [39]. The diazonium salt **45** was synthesized from aniline **44** by diazotization with *t*BuONO in BF_3_•OEt_2_ and MTBE as solvent [40]. Owing to its strong electron-withdrawing character, diazonium salt **45** efficiently promoted reactions with weakly nucleophilic alcohols **46** in a mixture of CD_3_CN:(CD_3_)_2_SO, to yield aryl–alkyl diazonium ethers **47** (Scheme 8). A subsequent TEMPO-promoted Pschorr cyclization of **47** furnished the tricyclic dibenzoxepanes **48** (Scheme 8), demonstrating, the utility of diazonium-mediated strategies for constructing oxygenated polycyclic frameworks.

### 3.4. Thermally Activated Pschorr Reaction

The benzoxanthene imide (BTI) framework is a key component in materials science and synthesis due to its use in textile dyes and applications such as organic photovoltaics, OLED/LEC and photodynamic therapy (PDT) [41,42].

**Scheme 9:**
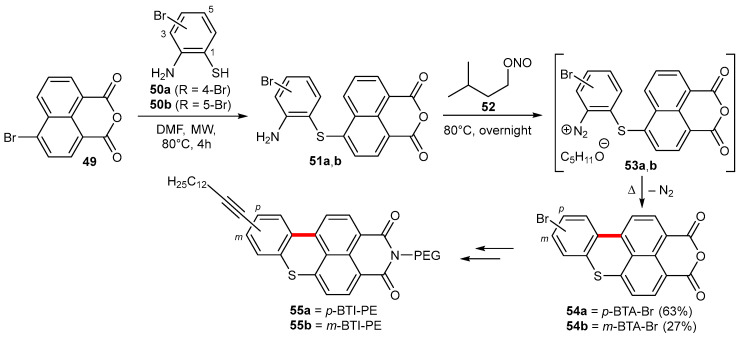
Synthesis of the brominated benzothioxanthene anhydride intermediates **54**, via a Pschorr cyclization, from aniline–thioether precursors **51**.

Synthesis of a series of BTI-based potential luminescence and liquid crystal materials **55** was undertaken, focusing on synthesis of the regioisomeric brominated benzothioxanthene anhydrides **54a** (*p*-BTA-Br) and **54b** (*m*-BTA-Br), as key intermediates for those materials [43]. A one-pot, microwave-assisted, thermal Pschorr cyclization was employed as the key step of the synthetic process (Scheme 9). Thus, brominated naphthalene anhydride **49** was coupled via a S_N_Ar with bromoaminobenzenethiols **50** to form the corresponding aniline–thioether intermediates **51**, which were subsequently converted in situ into the aryl diazonium salts **53** by treatment with isoamyl nitrite **52**. The diazonium salts **53** underwent a Pschorr radical ring closure, by thermal decomposition in the reaction medium, to furnish the polycyclic brominated benzothioxanthene anhydrides **54a**,**b**. Further synthetic steps converted the polycycles **54a**,**b** into the expected BTI-based liquid crystal composites **55a**,**b** (Scheme 9).

Polarized light optical microscopy (POM), differential scanning calorimetry (DSC), powder X-ray diffraction (PXRD) techniques and photophysical studies were used to confirm the structure and properties of the target BTIs **55**. In general, these compounds exhibited favorable emissive properties while maintaining the inherent advantages of LCs [43].

**Scheme 10:**
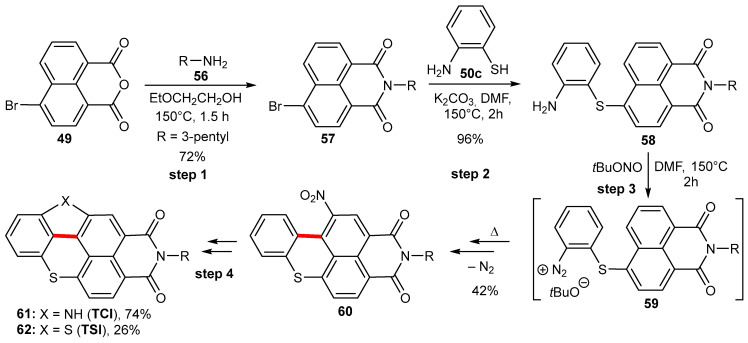
One-pot synthesis of the nitro-benzothioxanthene derivative **60** (NO_2_-BTI) mediated by a Pschorr cyclization, from the aniline–thioether precursor **58**.

In a further study, the same researchers developed a Pschorr-mediated synthetic strategy to obtain additional examples of *N*-annulated BTI derivatives **61** and **62** in a four-step sequence [44], improving a previous seven-step procedure [45]. The imidation of the brominated naphthalene anhydride **49** by treatment with 3-aminopentane **56** afforded the corresponding brominated imide **57**. Subsequently, nucleophilic coupling of **57** with the aminothiophenol **50c** (R = H) under basic conditions provided the key aniline–imidothioether precursor **58**. This precursor was then subjected to Pschorr cyclization mediated by tertbutyl nitrite (through thermal decomposition of the diazonium salt intermediate **59**) affording the unexpected nitro-benzothioxanthene imide (NO_2_-BTI) **60**. Compound **60** was then treated with either triphenylphosphine [46] or elemental sulfur [47] to deliver the corresponding *N*-annulated structure **61** (TCI) and the *S*-annulated structure **62** (TSI) respectively (Scheme 10) [44].

**Scheme 11:**
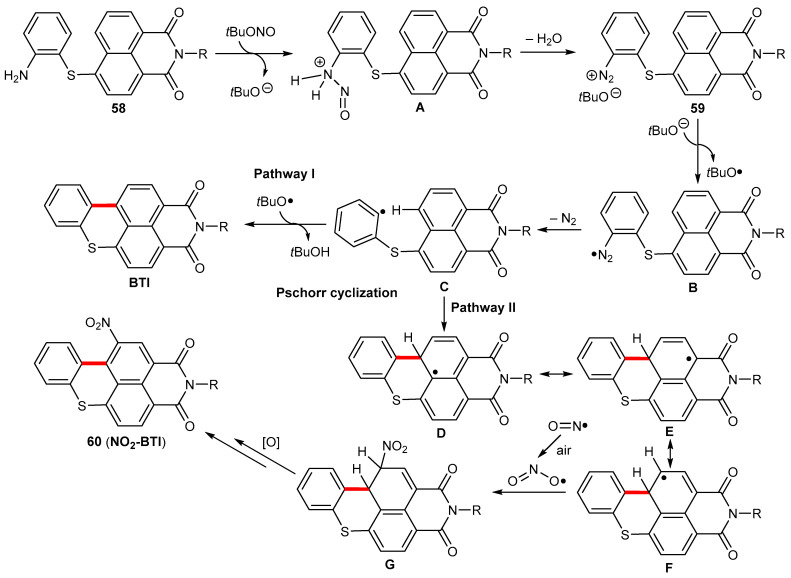
Plausible route to NO_2_-BTI during the thermal Pschorr cyclization of aniline–imidothioether **58**, promoted by *t*BuONO.

Formation of the nitro-derivative **60** (NO_2_-BTI) demonstrates that alkyl nitrite could act as nitrating agent under these conditions, which would offer advantages relative to conventional nitration, especially during scale-up processes [48]. A plausible mechanism for the formation of **60** (Scheme 11) starts with nitrosation of aniline–imidothioether **58** with *t*BuONO to give the ***N***-nitroso intermediate **A**, which upon dehydration is converted into the aryl diazonium salt **59**. Then the alkoxide (*t*BuO^−^) present in the reaction medium reacts with the diazonium group to afford the activated radical species **B**, promoting N_2_ extrusion and generating the key aryl radical **C**. This radical is delocalized into the aryl ring and can be trapped by NO_2_, formed in situ by oxidation of NO arising from the thermal decomposition of *t*BuONO. Finally, rearomatization of the nitro-intermediate **G** delivers the nitro-compound **60** (NO_2_-BTI).

## 4. Photoredox-Catalyzed Pschorr Reaction

### Synthesis of 6H-Benzo[c]chromenes

An organic photoredox Pschorr reaction for the synthesis of 6*H*-benzo[*c*]chromenes **8** was developed by using the organic dye eosin Y as catalyst under irradiation by green light-emitting diodes (LEDs) (Table 8) [49]. The amino-ethers **63** were synthesized by a nucleophilic aromatic substitution (S_N_Ar) reaction between nitrophenols **1** and bromo-derivatives **2** in the presence of K_2_CO_3_, and subsequent reduction of the nitro group with SnCl_2_•H_2_O. Then, in situ diazotization of amines **63** with aq *t*BuONO in HBF_4_ afforded the 2-(benzyloxy)benzenediazonium tetrafluoroborates **4** [19]. To continue with the synthesis of the target benzo[*c*]chromenes **8** an intramolecular Pschorr cyclization was undertaken. Optimization of the reaction conditions in terms of solvent, catalyst, irradiation, and concentrations established that the irradiation of the diazonium salts **4** with 36 W green LEDs in the presence of eosin Y in ACN gave the best yields of the cyclized products **8**, tolerating various functional groups (Table 8). Control experiments confirmed that both light potency and eosin Y play central roles in the efficacy of the intramolecular photoredox cyclization.

**Scheme 12:**
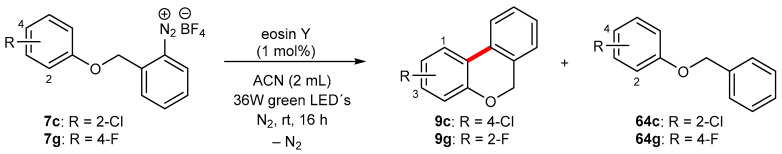
Synthesis of 6*H*-benzo[*c*]chromenes **9c**,**g** via eosin Y-promoted photoredox Pschorr reaction from diazonium salts **7c**,**g**.

Following the same procedures, diazonium salts **7c**,**g** were obtained as described previously in Scheme 1. Subsequently, these starting materials were subjected to the optimized reaction conditions affording the corresponding 4-chloro- and 2-fluoro-6*H*-benzo[*c*]chromenes **9c** and **9g**, respectively, along with the reduction products **64c**,**g** (Scheme 12).

**Scheme 13:**
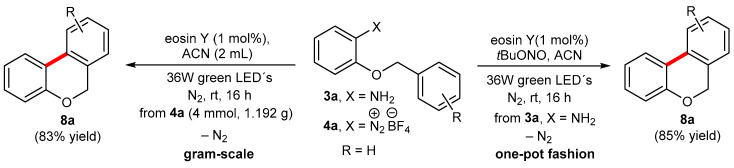
Synthesis of 6*H*-benzo[*c*]chromene **8a** both in gram scale and in a one-pot fashion via eosin Y-promoted photoredox Pschorr reaction.

Additionally, the robustness of this photoredox protocol was assessed by a gram-scale synthesis, as well as a one-pot synthesis of the 6*H*-benzo[*c*]chromene (**8a**) (R = H). In this strategy, the diazonium salt **4a** was generated in situ by addition of *t*BuONO to the aniline **3a** and subsequent irradiation of the reaction mixture under the established photoredox conditions afforded the expected product **8a** as shown in Scheme 13.

**Scheme 14:**
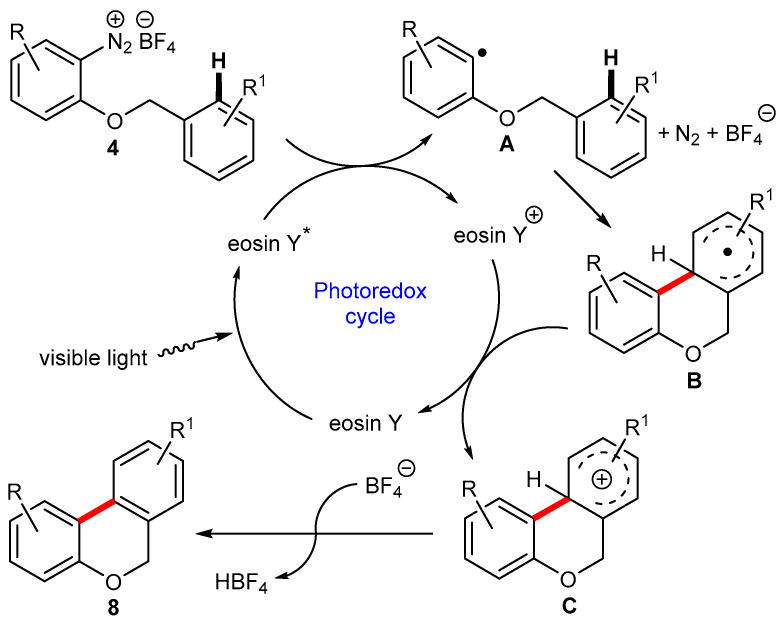
Plausible mechanism for the intramolecular photoredox Pschorr cyclization of the diazonium salts **4**.

A plausible mechanism for the intramolecular photoredox Pschorr cyclization of the diazonium salts **4** is depicted in Scheme 14. It involves reduction of **4** to the aryl radical **A** by the excited photoredox catalyst eosin Y* via single-electron transfer, while eosin Y is oxidized to eosin Y^+^. Then, intramolecular cyclization of **A** generates the radical intermediate **B**, which after oxidation with eosin Y^+^ regenerates the original eosin Y, completing the catalytic cycle and providing the aryl cation **C**, which subsequently is deprotonated by the BF_4_^–^ counterion to afford the expected product **8** [49].

## 5. Pschorr-Inspired Benzannulations and Extensions—Formation of Spirocyclic Systems

### 5.1. Intramolecular Benzannulations

#### 5.1.1. Synthesis of Fused-Hydro-Aromatic Systems by Intramolecular Ring Closure

**Scheme 15:**
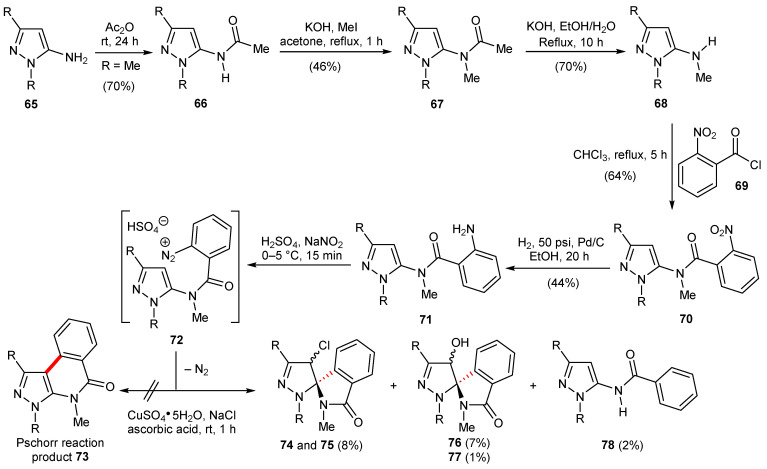
Synthesis of spirocyclic chlorinated and hydroxylated pyrazole derivatives **74**–**77** from the aniline precursor **71** by a Cu-catalyzed cyclization.

Pyrazole-based heterocycles are important in medicinal and synthetic chemistry due to their broad range of biological activities and versatility as key intermediates for assembly of complex molecular architectures [50,51]. A synthetic sequence aimed at the synthesis of tricyclic pyrazolo-derivative **73** via a Pschorr ring closure began with the acylation of 1,3-dimethyl-1*H*-pyrazol-5-amine **65** using acetic anhydride to afford the corresponding acetamide **66** (Scheme 15) [52]. Subsequent *N*-methylation under basic conditions with methyl iodide furnished the *N*-methyl-derivative **67**, which upon hydrolysis provided the *N*-methylated 5-aminopyrazole **68**, whose acylation with 2-nitrobenzoyl chloride **69** afforded the 2-nitrobenzamide **70**. Catalytic hydrogenation of **70** afforded the corresponding 2-amino-*N*-methylbenzamide **71**, and subsequent diazotization of **71** in the presence of sulfuric acid generated the diazonium hydrogen sulfate **72**, which was then subjected to a Pschorr cyclization in the presence of copper sulfate, sodium chloride, and ascorbic acid. Surprisingly this transformation did not afford the expected tricyclic pyrazolo-derivative **73**; instead, a mixture of the non-separable chlorinated spirocyclic epimers **74**/**75**, along with separable hydroxyl spirocyclic epimers **76**/**77**, and the benzamide **78** were obtained (Scheme 15).

**Scheme 16:**
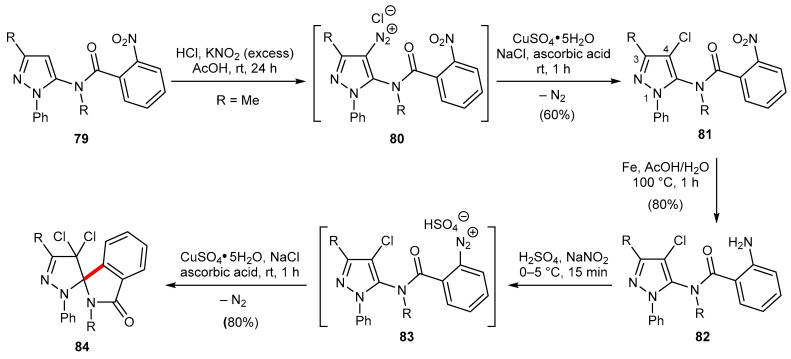
Synthesis of spirocyclic pyrazole derivative **84** from the aniline precursor **82** via a Cu-catalyzed Pschorr-type cyclization.

To further explore the scope and synthetic potential of the transformation described in Scheme 15, a complementary route was developed starting from the 2-nitrobenzamide precursor **79** (Scheme 16) [52]. Thus, treatment of **79** with hydrochloric acid and a large excess of potassium nitrite in acetic acid directly afforded the nitro-diazonium salt **80** [53]. Subsequent in situ conversion of intermediate **80** into the corresponding 4-chloropyrazole derivative **81** was achieved using CuSO_4_•5H_2_O, NaCl, and ascorbic acid as catalytic system, thus blocking the 4-position of the pyrazole moiety in **81**. Reduction of the nitro group in **81** with iron in an acetic acid–water mixture afforded the aniline derivative **82**, which was further subjected to diazotization to furnish the intermediate diazonium salt **83**. Under identical Cu(II)/ascorbate-mediated radical conditions to those applied for the diazonium salt **80**, a Pschorr-type cyclization of **83** delivered the dichlorinated spirocycle **84** (Scheme 16).

**Scheme 17:**
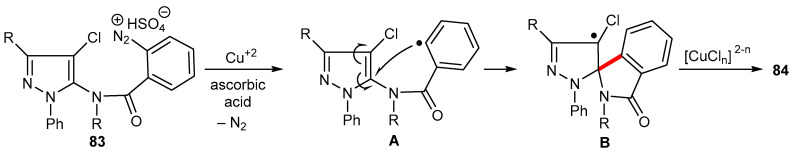
Plausible mechanism for the synthesis of the spirocyclic pyrazole derivative **84** via a Cu(II)-mediated Pschorr-type cyclization.

A plausible mechanism for the Cu(II)-catalyzed formation of the spirocycle **84** from diazonium salt intermediate **83** (Scheme 17) involves generation of the radical species **A** and subsequent ring closure to form radical species **B** which reacts with cuprous chloride to yield the dichlorinated spirocycle **84**. The key role of the ascorbic acid is to reduce Cu(II) to Cu(I) which, in turn, reduces the diazonium ion **83** to a diazenyl radical. The latter intermediate decomposes to dinitrogen (N_2_) and the phenyl radical **A** species [52].

**Scheme 18:**
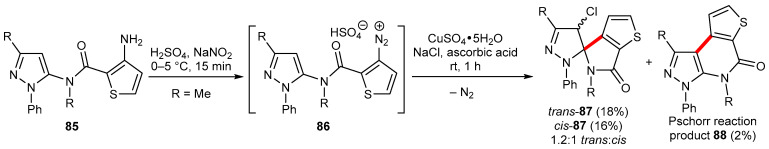
Cu(II)-mediated Pschorr-type cyclization for the synthesis of pyrazole derivatives **87** and **88** from the amino-precursor **85**.

**Scheme 19:**
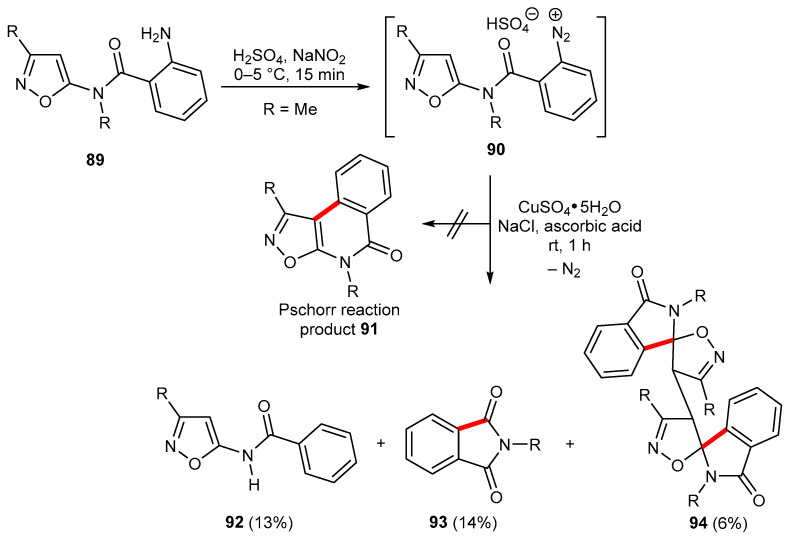
Cu(II)-mediated Pschorr-type cyclization leading to isoindoline-1,3-dione **93** (R = Me) and isoxazole derivative **94** (R = Me) from the aniline precursor **89**.

Further studies on spirocyclization via Pschorr-type reaction examined the influence of (hetero)aromatic substituents tethered through an amide linker on the cyclization outcome (Scheme 18 and Scheme 19) [52]. Accordingly, amines **85** and **89** were subjected to diazotization with sulfuric acid and sodium nitrite, and the resulting diazonium salts **86** and **90** were exposed to CuSO_4_•5H_2_O, NaCl, and ascorbic acid to promote aryl radical formation. Under these conditions, diazonium salt **86** underwent a highly selective 5-*exo*-*trig* cyclization to give the spiro-system **87** (34%) as a *trans*/*cis* mixture, accompanied by trace amounts of the expected tricyclic Pschorr product **88** arising from a competing 6-*endo*-*trig* process (Scheme 18).

With diazonium salt **90**, the Pschorr reaction product **91** (the 6-*endo*-*trig* mode) was not observed; instead, a 5-*exo*-*trig* cyclization prevailed, yielding methylisoindoline-1,3-dione **93** and the *bis*-spiro compound **94** (Scheme 19) [52]. In parallel, a competing 1,5-hydrogen shift from the *N*-methyl group in **90** accounted for the formation of the secondary amide **92** [54].

The products obtained in these transformations (Scheme 18 and Scheme 19) signify the interplay of radical cyclization kinetics, the electronic nature of the (hetero)aromatic acceptor, and the competing 1,5-H shift processes [54]. While the 5-*exo-trig* closure is generally favored, the alternative 6-*endo-trig* or hydrogen-shift pathways also operate depending on the substitution pattern. In this synthetic design, the Pschorr-type cyclization proved to be a valuable method for the synthesis of spirocyclic architectures **87** and **94**.

**Scheme 20:**
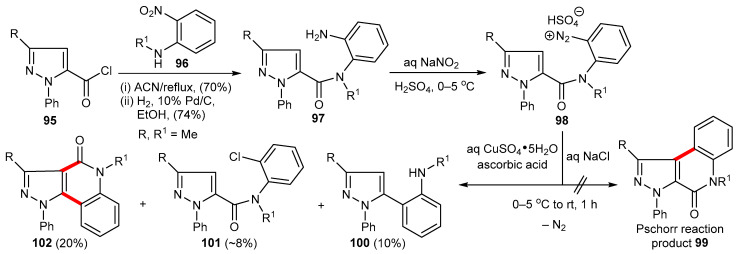
Cu(II)-mediated Pschorr-type cyclization forming the tricyclic dihydro-4*H*-pyrazolo[4,3-*c*]quinolin-4-one **102** (R,R^1^ = Me), from the aniline precursor **97**.

In another study, the same authors investigated substrates with an inverted amide bridge between the pyrazole and phenyl rings [55]. The key -CONCH_3_-based diazonium salt intermediate **98** was synthesized from the acyl chloride **95** and the *o*-nitroaniline **96** followed by the catalytic reduction of the nitro group to give **97**, which after diazotization yielded the diazonium hydrogen sulfate **98** (Scheme 20). Intermediate **98** was then subjected to the Pschorr reaction, employing the redox system comprising CuSO_4_, NaCl, and ascorbic acid. This resulted in three products **100**–**102** instead of the expected tricyclic product **99** (Scheme 20).

**Scheme 21:**
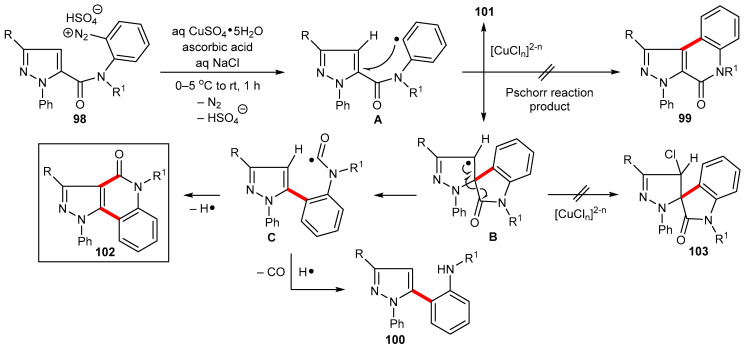
Plausible mechanism for the Cu(II)-mediated Pschorr-type cyclization affording a mixture of products including pyrazolo[4,3-*c*]quinolin-4-one **102** as the main component.

Formation of tricyclic dihydro-4*H*-pyrazolo[4,3-*c*]quinolin-4-one **102**, a compound exhibiting affinity for the benzodiazepine receptor, via the Pschorr-type reaction is noteworthy. The suggested mechanism for its formation as a major component is depicted in Scheme 21, and involves intramolecular rearrangements involving a 1,4-transfer of the pyrazolyl group (pathway from **A** to **C**), followed by a Pschorr-type ring closure of the carbamoyl radical **C**.

##### Biological Studies

Compound **102** displaced [^3^H]flunitrazepam from its benzodiazepine receptor site in bovine brain membranes (IC_50_ = 3.4 ± 0.2 μM), as well as exhibited antileukemic activity against HL-60 cell line inhibiting about 40% cell growth at 10 μM. These findings point to it being a potential antiproliferative agent, as well as a relevant pharmaceutical for CNS-related therapeutic research [55].

### 5.2. Aza-Pschorr-Type Reactions

**Figure 2:**
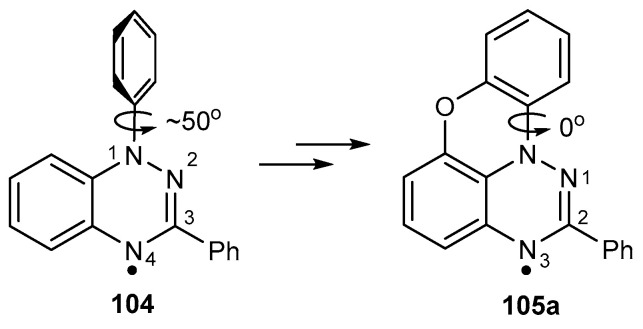
The non-planar *N*-centered Blatter radical **104** and its planar Blatter analog **105a**.

The *N*-centered Blatter radical **104** is notable for its exceptional thermodynamic and kinetic stability, mainly due to extensive π-delocalization [56,57]. This radical and its derivatives have gained attention for their electrochemical properties and potential applications in the design of functional materials for molecular electronics, spintronics and photoconductive liquid crystals, among others [58,59,60]. The large torsion angle between the two π planes caused by rotation of the N-1-aryl group in radical **104** affects its molecular packing which is unfavorable for induction of liquid crystalline and semiconductive properties (Figure 2) [61]. This then calls for methods to convert the non-planar radical **104** to a planar radical analog **105a**. Since previous anionic methods for the construction of the triazinephenoxazine core of the planar radical **105a** were limiting, a radical-mediated aza-Pschorr-type cyclization approach was developed to synthesize **105a** and its C(10)-functionalized derivatives [62].

The key amino-ether derivatives **108** were synthesized from 8-fluoro-3-phenyl-benzo[*e*][1,2,4]triazine (**106**) through an S_N_Ar with *o*-amino/-nitrophenolic compounds **107** in the presence of NaH/DMSO to afford the corresponding amino-ethers **108**. After optimization of the reaction conditions, treatment of the amino-ethers **108** with *t*BuONO in chlorobenzene as solvent generated the corresponding diazonium salts **109**, which under thermal dediazoniation furnished the planar Blatter radicals **105**/**110** (Table 9).

To shed light on the role of substituents on the electronic properties, namely stability, redox behavior, absorption spectra and spin distribution, synthesis of a larger series of C(10)-substituted derivatives of **105** was undertaken, starting with diversely substituted triazine-precursors **108** (synthesized according to Table 9). Cyclization via aza-Pschorr type (methods A/B via diazonium salts) was compared with photochemical (method C) and Bu_3_SnH-assisted radical cyclization (method D, Table 10) [62,63,64,65,66]. Additionally, functional group transformations of the C(10)-amino/-nitro/-iodo derivatives were performed in some cases, starting from previously obtained planar radicals **105**.

**Figure 3:**
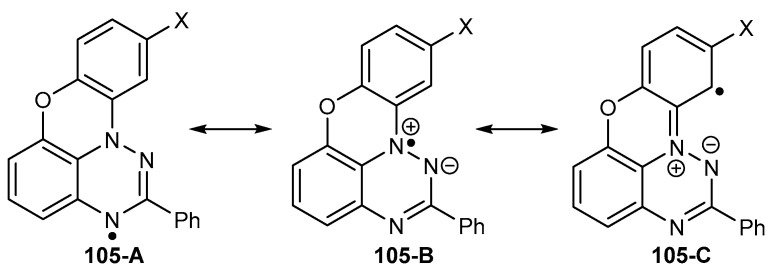
Three resonance forms of the planar Blatter radicals **105**. The polar form **105-B** is responsible for spin delocalization into the “upper” benzene ring.

Further experiments and analytical techniques such as cyclic voltammetry, electron paramagnetic resonance spectroscopy, and correlation analysis were employed to characterize the planar Blatter radicals **105**. The results indicated that electron-donating substituents C(10)–X lower the excitation energies and redox potential, and increase radical stability by increasing electron spin delocalization (Figure 3) and decreasing the N–H bond dissociation energy [66].

Further studies were aimed at the synthesis of π-extended liquid crystalline radicals **115**. For this purpose, the intramolecular aza-Pschorr-type cyclization was employed as a key step to obtain the target radicals **115** [63]. The synthetic sequence started with the nitro-hydrazide **111**, which upon reduction, cyclization, and oxidation afforded the 8-fluorobenzo[*e*][1,2,4]triazine **102** which was reacted with the sodium salt **107** in an S_N_Ar fashion to afford the triazolo-aniline **112**.

**Scheme 22:**
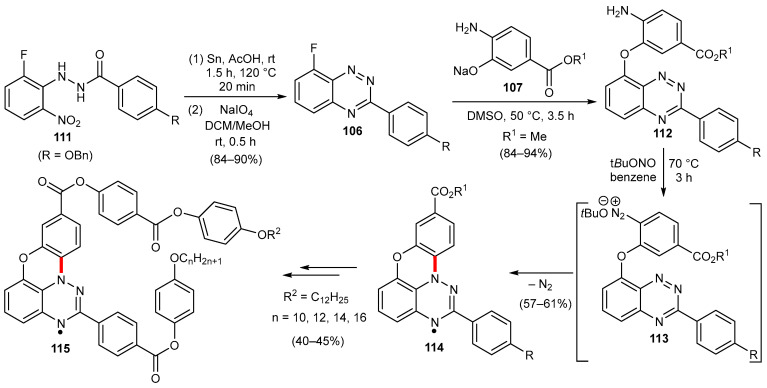
Synthesis of the planar radical intermediate **114** via a thermal aza-Pschorr-type cyclization from triazolo-aniline precursor **112**.

Subjecting the triazolo-aniline **112** to diazotization with *t*BuONO followed by a homolytic dediazoniation furnished the planar Blatter radical intermediate **114**, which was transformed into the target bent-core and π-extended liquid crystalline radicals **115** (Scheme 22).

**Figure 4:**
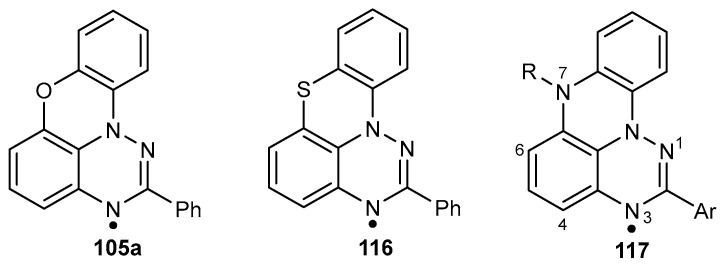
Structures of the *N*-centered O-, S- and N-peri-annulated planar Blatter radicals **105a**, **116** and **117**, respectively.

The aza-Pschorr-type cyclization method was further utilized as a practical synthetic strategy for the construction of *N*-centered O- and S-peri-annulated planar Blatter radicals **105a** and **116** [67]. Further studies aimed at developing a synthetic route to their *N*-centered N-peri-annulated radical analogs **117** (Figure 4) [68]. While the chalcogen atoms in O-peri- **105a** and S-peri-annulated **116** radicals are divalent, the N(7) nitrogen atom in the N-peri-annulated radical analogs **117** is trivalent, for which the R-substituent connected to this atom could be used to tune the electronic system in **117**.

**Scheme 23:**
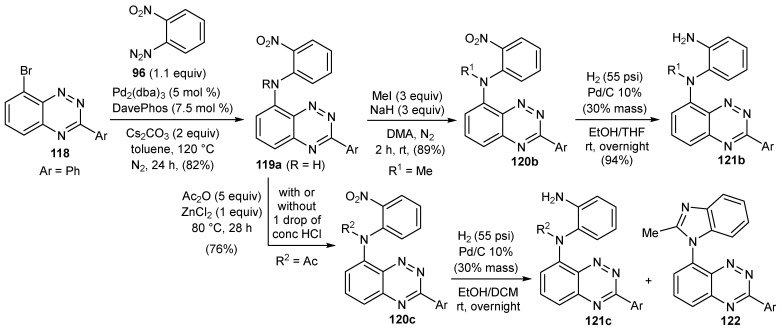
Synthesis of the *N*-methylaniline-derivative **121b** as precursor for the N-peri-annulated planar radical **117b** via an aza-Pschorr-type cyclization.

Two complementary synthetic approaches, namely the aza-Pschorr-type cyclization and photocyclization, were considered for the synthesis of the N-peri-annulated radicals **117** [68]. Since several attempts to obtain the N-R nitro-precursor **119a** (R = H) by aza-Pschorr-type cyclization failed, a Pd-catalyzed C−N cross-coupling reaction of 8-bromo-[1,2,4]triazine **118** with *o*-nitroaniline **96** in the presence of Pd_2_(dba)_3_/Cs_2_CO_3_ was used instead, to produce the desired intermediate product **119a** as red crystals (Scheme 23). *N*-methylation using MeI/NaH afforded the *N*-methyl nitro-derivative **120b** and catalytic reduction of this compound with H_2_/Pd/C afforded the corresponding the *N*-methylamino-derivative **121b**, while acetylation of nitroaniline **119a** with Ac_2_O/ZnCl_2_ generated the *N*-acetyl derivative **120c**.

Attempts to reduce the nitro group, in absence of HCl, afforded an inseparable mixture of the *N*-acetyl-aniline **121c** and the imidazole side-product **122** (Scheme 23). When the reduction of **120c** was carried out in the presence of catalytic HCl, the side-product **122** was isolated in near-quantitative yield.

**Scheme 24:**
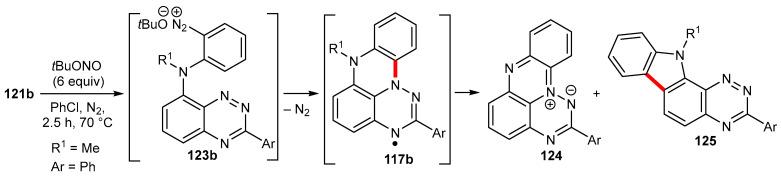
Reaction products **124** and **125** isolated from the thermal aza-Pschorr-type cyclization of the *N*-methylamino-radical precursor **121b**.

Using **121b** as precursor, the synthesis of the target N-peri-annulated planar radical **117b** was attempted by employing an aza-Pschorr-type cyclization, via treatment of the *N*-methylamino precursor **121b** with *t*BuONO, followed by thermal decomposition of the diazonium salt intermediate **123b**. However, **121b** did not lead to the N-peri-annulated radical **117b** (R = Me); instead, two products, namely the zwitterionic species **124** and the fused carbazole **125**, were isolated (Scheme 24).

Although the expected N-peri-annulated radical **117b** was not isolated from the thermal aza-Pschorr-type cyclization, the isolation of the zwitterionic species **124** indicated that **117b** must have been formed as a short-lived intermediate, suffering a demethylation under the thermal conditions. Similar results were obtained from the photochemical reaction, which also converted the nitro-compound **120b** into the zwitterionic species **124** [68].

**Scheme 25:**
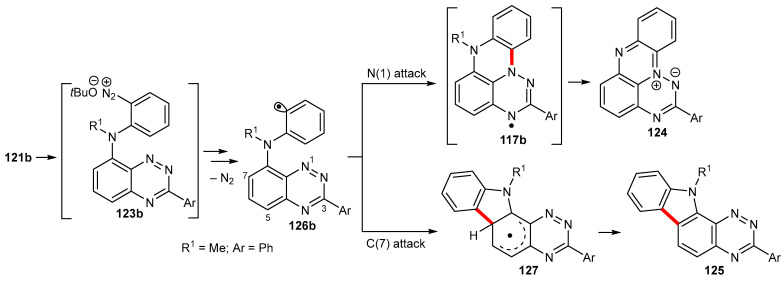
Proposed mechanistic sequence for the formation of the zwitterionic species **124** and fused carbazole derivative **125**, via aza-Pschorr-type cyclizations.

Formation of the zwitterionic species **124** and the fused carbazole derivative **125** could proceed through two competing processes involving the initial formation of the *N*-methylamino-radical species **117b**, which could either cyclize at the N(1) position, leading to the expected N-peri-annulated radical **117b**—whose decomposition, accompanied by demethylation, leads to **124**—or alternatively cyclize at the C(7), generating the tetracyclic radical **127**, which is transformed into **125** after homolytic dehydrogenation and re-aromatization (Scheme 25).

DFT calculations were in concert, showing that the activation energy required for N(1) attack to form species **117b** is lower (by 1.35 kcal mol^−1^) than that for the C(7) attack to form species **127**. The difference in the activation energies correlates with the observed relative yields of the zwitterionic product **124** and the fused carbazole derivative **125** [68].

### 5.3. Organoboronic Acids as Novel Precursors for Pschorr-Type Cyclization

#### Synthesis of Fluorenones and Dibenzofuranes

Whereas the iron- or copper-catalyzed Pschorr cyclization of arenediazonium salts constitutes a classical method to access hetero-PAHs, including fluorenones and dibenzofurans, development of newer methods that avoid the use of diazonium salts has received recent attention. In this connection organoboronic acids were introduced as novel precursors for borono-Pschorr-type cyclization [69].

The synthetic sequence for the substituted fluorenones **31** started with preparation of the brominated diarylketones **129** from a three-step procedure (i–iii) starting with the aryl halides **128** (Table 11). Subsequent treatment of bromo-ketones **129** with pinacol borane catalyzed by PdCl_2_ and reaction with KHF_2_ gave the corresponding potassium trifluoroborate salts **130**, as key intermediates for the borono-Pschorr-type cyclization. Reaction of trifluoroborates **130** with catalytic silver nitrate and stoichiometric potassium persulfate in aqueous medium [70] afforded the target fluorenones **31** as described in Table 11.

**Scheme 26:**
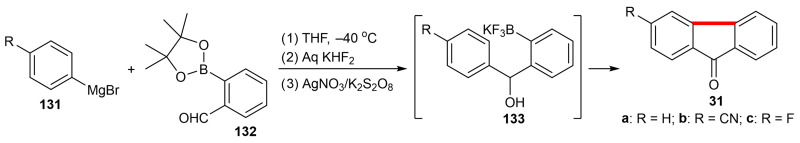
Alternative synthetic route for fluorenones **31** employing *o*-formyl pinacol boronate ester **132**.

Alternatively, fluorenones **31** were synthesized by reaction of aryl Grignard reagents **131** with *o*-formyl pinacol boronate ester **132** followed by aq KHF_2_, generating the alcohol intermediates **133**. The hydroxy-trifluoroborate salts **133**, upon treatment with AgNO_3_/K_2_S_2_O_8_, afforded fluorenones **31** (Scheme 26) [69].

This protocol was then extended to the synthesis of dibenzofurans **136**, directly from the key organoboronic acids **135**. This procedure involved the previous preparation of **135** from a three-step procedure (i–iii) starting with the *o*-iodo- or *o*-bromo-diarylethers **134**, and subsequently subjecting organoboronic acids **135** to the established AgNO_3_/K_2_S_2_O_8_-catalyzed reaction conditions, to yield the benzofurans **136** (Table 12) [69].

## 6. Summary and Future Outlook

The present review highlights and underscores the recent developments in the Pschorr reaction and its offshoots, and their application in the synthesis of PAHs and hetero-PAHs. Following the synthesis of the key diazonium salts, dediazoniation is achieved by a variety of methods, namely by metal catalysis, metal-free, thermally, or by photoredox chemistry, to form the aryl radical intermediates. These approaches are utilized to develop more efficient synthetic routes to important small-molecule bioactive compounds. The Pschorr-inspired benzannulation methods and their off-shoots, namely the aza-Pschorr and the borono-Pschorr cyclizations, are noteworthy areas deserving further expansion. Looking globally, it is highly notable how this classical method for aryl radical generation and annulation, starting from the diazonium salts, has inspired the development of numerous more modern methods and off-shoots that provide shorter, more efficient synthetic routes to sought-after bioactive compounds.

## Figures and Tables

**Table 1:** Synthesis of 6*H*-benzo[*c*]chromenes **8** via a Pd(OAc)_2_-catalyzed Pschorr reaction from diazonium salts **4**.

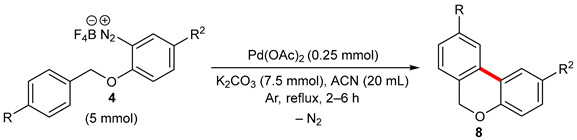				
**Entry**	**Compound**	**R**	**R^2^**	**Yield (%)**
1	**8a**	H	H	75
2	**8b**	Br	H	68
3	**8c**	Cl	H	70
4	**8d**	Me	H	76
5	**8e**	H	Me	75
6	**8f**	Me	Me	80
7	**8g**	Cl	Me	72
8	**8h**	Br	Me	71
9	**8i**	H	*t*Bu	78
10	**8j**	Me	*t*Bu	83
11	**8k**	Cl	*t*Bu	76
12	**8l**	Br	*t*Bu	72
13	**8m**	OMe	H	48
14	**8n**	H	CO_2_Et	83

**Table 2:** Synthesis of 6*H*-benzo[*c*]chromenes **9** via a Pd(OAc)_2_-catalyzed Pschorr reaction from diazonium salts **7**.

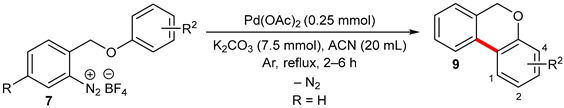			
**Entry**	**Compound**	**R^2^**	**Yield (%)**
1	**9a**	H	65
2	**9b**	4-F	52
3	**9c**	4-Cl	50
4	**9d**	4-Br	68
5	**9e**	4-Me	58
6	**9f**	2-Me	57

**Table 3:** Synthesis and anti-melanogenic activity of 4,5-didehydroguadiscines **18** against B16 melanoma 4A5 cells through a CuCl-catalyzed Pschorr reaction.

Entry	Compound	R	R^1^	R^2^	Yield from 16 (%)	IC_50_ (µM) ^a^
1	**18a**	OCH_2_O		MeO	--	4.9
2	**18b**	OCH_2_O		H	25	6.7
3	**18c**	MeO	H	H	30	5.0
4	**18d**	H	MeO	H	20	4.0
5	**18e**	H	H	H	29	3.1
6	**18f**	MeO	H	MeO	61	7.5
	**Arbutin**	--	--	--	--	174

^a^ The half-maximal inhibitory concentration (IC_50_) of each compound was determined after 72 h of treatment with graded doses (1, 3, 10, and 30 μM). Untreated cells served as control, and cell viability was evaluated using the MTT assay.

**Table 4:** Synthesis of fluorenone derivatives **31** via a gold-catalyzed Pschorr cyclization using diazonium salts **30**.

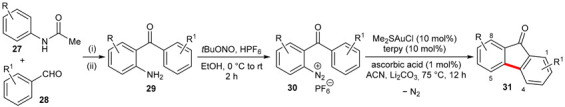				
**Entry**	**Compound**	**R**	**R^1^**	**Yield from 30 (%)**
1	**31a**	H	H	74
2	**31b**	7-Me	H	74
3	**31c**	7-OMe	H	68
4	**31d**	7-F	H	45
5	**31e**	7-Cl	H	33
6	**31f**	H	3-F	71
7	**31g**	H	3-Br	79
8	**31h**	H	3-I	51
9	**31i**	H	3-CF_3_	53
10	**31j**	H	3-Me	62
11	**31k**	H	3-*t*Bu	57
12	**31l**	H	3-(4-MeOC_6_H_4_)	92
13	**31m**	H	3-Mesityl	52
14	**31n**	H	3-(4-*t*BuC_6_H_4_)	60
15	**31o**	H	3-(1-Naphthyl)	54
16	**31p**	H	3-(4-CF_3_C_6_H_4_)	59
17	**31q**	H	3-(4-NO_2_C_6_H_4_)	49
18	**31r**	6-Me	3-Cl	66
19	**31s**	6-Me	3-Br	54
20	**31t**	6-Me	3-NO_2_	55
21	**31u**	7-Me	3-Cl	58
22	**31v**	7-OMe	3-Cl	53
23	**31w**	7-Cl	1-F	29
24	**31x**	7-Cl	3-Cl	20
25	**31y**	6-Me	3-OMe	85
26	**31z**	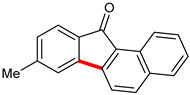		61
	**31z′**	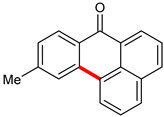		12

(i) Pd(TFA)_2_ (5 mol%), TBHP, PhMe, 90 °C, 24 h; (ii) MeOH, HCl, reflux, 4 h.

**Table 5:** Antimycobacterial activity of the mixture of phenanthrene compounds **40a**/**40b**.

Entry	Strain	40a/40b (MIC µM)	Streptomycin (MIC µM)
1	*M. smegmatis*	>991.50	2.68
2	*M. bovis*	991.50	0.67
3	*M. szulgai*	>991.50	1.30
4	*M. gastri*	>991.50	0.17
5	*M. simiae*	>991.50	2.68
6	*M. tuberculosis* (H37Ra)	>991.50	0.84

**Table 6:** Product distribution resulting from both Pschorr and dediazoniation reactions of diazonium salts **30** in the presence of [BMIM][TfO].

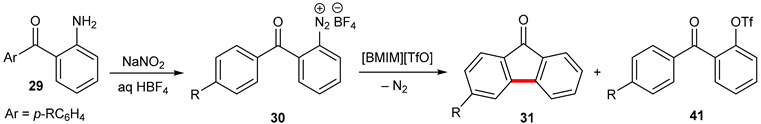					
**Entry**	**R**	**T (°C)**	**Time (h)**	**Yield Ratio ^a^**	**Total Yield of 31 and 41 (%) ^a^**
1	H	50	6	**31a**:**41a** (66:34)	79
2	Me	80	1.5	**31b**:**41b** (58:42)	84
3	MeO	80	2	**31c**:**41c** (53:47)	78
4	Cl	80	1	**31d**:**41d** (42:58)	74

^a^ The yield ratio was determined by ^1^H NMR analysis.

**Table 7:** Product distribution from dediazoniation of salts **30** in [BMIM][Tf_2_N].

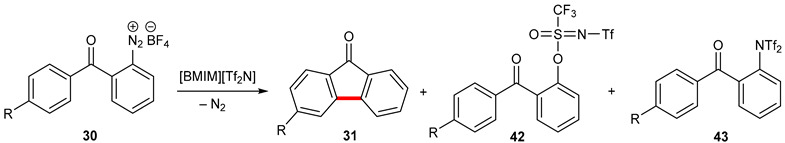					
**Entry**	**R**	**Temp (°C)**	**Time (h)**	**Yield Ratio ^a^**	**Total Yield of 31, 42, and 43 (%) ^a^**
1	H	50	6	**31a**:**42a**:**43a** (85:15:<1)	91
2	Me	80	1.5	**31b**:**42b**:**43b** (75:24:1)	94
3	MeO	80	2	**31c**:**42c**:**43c** (72:27:1)	84
4	Cl	80	1	**31d**:**42d**:**43d** (78:22:<1)	72

^a^ The yield ratio was determined by ^1^H NMR analysis.

**Table 8:** Synthesis of 6*H*-benzo[*c*]chromenes **8** via an eosin Y-promoted photoredox Pschorr reaction from 2-(benzyloxy)benzenediazonium tetrafluoroborates **4**.

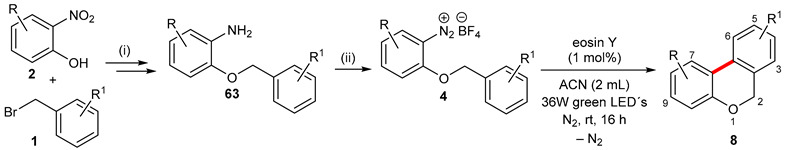				
**Entry**	**Compound**	**R**	**R^1^**	**Yield from 63 (%)**
1	**8a**	H	H	90
2	**8b**	H	5-Me	95
3	**8c**	H	3-Me	48
4	**8d + 8′-d**	H	4-Me + 6-Me	(49:51) 98
5	**8e + 8′-e**	H	4-MeO + 6-MeO	(22:78) 73
6	**8f**	H	5-Cl	30
7	**8g**	8-Me	5-Me	88
8	**8h**	8-Me	H	90
9	**8i**	8-Me	3-Me	64
10	**8j + 8′-j**	8-Me	4-Me + 6-Me	(50:50) 77
11	**8k + 8′-k**	8-Me	4-MeO + 6-MeO	(24:76) 73
12	**8l**	8-Cl	H	72
13	**8m**	8-Cl	5-Me	57
14	**8n**	9-F	H	80
15	**8o**	8-NO_2_	H	29

(i): **1** (1.0 equiv), **2** (1.0 equiv), K_2_CO_3_, ACN, then, the obtained nitrobenzyloxy derivative (1.0 equiv), SnCl_2_•H_2_O, AcOEt; (ii): **63** (1.0 equiv), aq *t*BuONO (1.5 equiv), HBF_4_ (1.5 equiv), 5 °C.

**Table 9:** Synthesis of planar Blatter radicals **105**/**110** via a thermal aza-Pschorr-type cyclization from diazonium salts **109**.

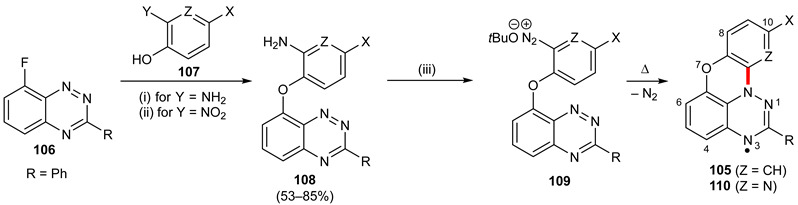			
**Entry**	**Compound**	**X**	**Yield from 108 (%)**
1	**105a**	H	57–64
2	**105b**	CO_2_Me	57–64
3	**105c**	CN	68
4	**105d**	NO_2_	40–55
5	**110**	H	50–60

(i) 60% NaH, DMSO, 100 °C, 1 h. (ii) 60% NaH, DMSO, 100 °C, 1 day; then H_2_ (2 bar), 10% Pd/C THF/EtOH, rt, overnight. (iii) amine **108** (0.25 mmol), PhCl (2 mL), *t*BuONO, rt to 70 °C, 15 to 45 min.

**Table 10:** Synthesis of diversely C(10)-substituted planar Blatter radicals **105** via aza-Pschorr-type cyclizations and comparison with other cyclization methods starting from radical precursors **108**.

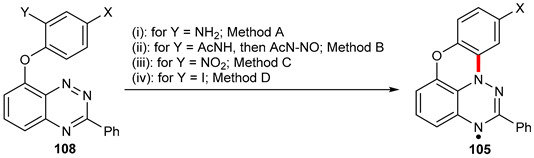				
**Entry**	**Compound**	**X**	**Method**	**Yield (%)**
1	**105a**	H	A (B)	57–64 (64–80)
2	**105b**	CO_2_Me	A (B)	48–55 (64−80)
3	**105c**	CN	A (B)	68 (60)
4	**105d**	NO_2_	A	40–55
5	**105e**	CF_3_	A	62
6	**105f**	OMe	A	52
7	**105g**	F	A	45
8	**105h**	Cl	A	55
9	**105i**	Br	A	18
10	**105j**	COMe	D	47–52
11	**105k**	CO_2_H	From **105b**	90–95
12	**105l**	NH_2_	From **105d**	82
13	**105m**	NHAc	From **105l**	85
14	**105n**	I	A	42–47
15	**105o**	OBn	A (C)	29 (35–49)
16	**105p**	Ph	From **105n**	61
17	**105q**	2-thienyl	From **105n**	53
18	**105r**	C≡CPh	From **105n**	50
19	**105s**	OAc	From **105j**	15
20	**105t**	NHCOCF_3_	From **1051**	73
21	**105u**	NHCO_2_Me	From **105l**	68

(i) Method A: (a) *t*BuONO, rt to 70 °C, 15 min, then 45 min at 60–70 °C. (ii) Method B: PhCl, Zn powder, 1 h at 60 °C. (iii) Method C: 300 W halogen lamp, DCM. (iv) Method D: Bu_3_SnH or TMS_3_SiH, cat. AIBN, toluene, 4 h at 80 °C.

**Table 11:** Synthesis of substituted fluorenones **31** from trifluoroborates **130** via an AgNO_3_/K_2_S_2_O_8_-catalyzed borono-Pschorr-type cyclization.

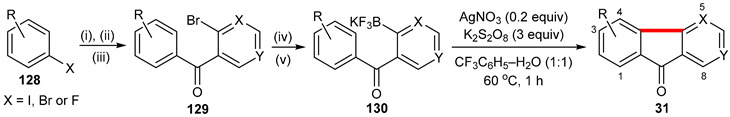					
**Entry**	**Compound**	**R**	**X**	**Y**	**Yield from 130 (%)**
1	**31a**	H	CH	CH	41
2	**31b**	3-CN	CH	CH	55
3	**31c**	3-F	CH	CH	50
4	**31d**	3-OMe	CH	CH	43
5	**31e**	3-CO_2_Me	CH	CH	67
6	**31f**	1,3-diCF_3_	CH	CH	61
7	**31g**	2,3,4-tri-F	CH	CH	71
8	**31h**	H	N	CH	64 (1.4:1; **31h**:**31i**)
9	**31i**	H	CH	N	

(i): *i*PrMgCl or *n*BuLi (1.0–1.05 mmol), THF (1–10 mL), −78 °C to 0 °C, 30 min to 3 h; (ii): *o*-bromobenzaldehyde/bromoformylpyridines (0.9–1.05 mmol), −78 °C to rt, 30 min to 3 h; (iii): MnO_2_ (10.0 mmol), DCM; (iv): pinacol borane (1.5 mmol), MeOH (2 mL), PdCl_2_(ACN)_2_ (0.02 mmol), SPhos (0.08 mmol), TEA (1.5 mmol), *p*-dioxane (0.6 mL), MWI (110 °C), (1–2 h); (v): KHF_2_ (3 equiv), rt, 30 min.

**Table 12:** Synthesis of substituted dibenzofurans **136** from organoboronic acids **135** via AgNO_3_/K_2_S_2_O_8_-catalyzed Pschorr-type cyclization.

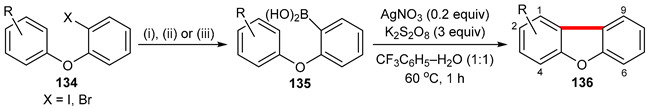			
**Entry**	**Compound**	**R**	**Yield (%)**
1	**136a**	2-CF_3_	73
2	**136b**	2-F	69
3	**136c**	2-CO_2_Me	57
4	**136d**	H	65
5	**136e**	1-OMe	41 (1:5; **136e**:**136f**)
6	**136f**	3-OMe	

(i): for **134a**,**b**, X = I: **134a,b** (1.0 equiv) in PhH (10 mL), (*i*PrO)_3_B (1.2 equiv) in THF-toluene (3.4 mL) at −78 °C, *n*BuLi (1.2 equiv in hexanes), −78 °C to −20 °C, 60 min, then 2N HCl (3 mL); (ii): for **134c**, X = I: **134c** (1.0 equiv), (*i*PrO)_3_B (1.9 equiv) in THF (1.4 mL) at 0 °C, *i*PrMgCl•LiCl (1.1 equiv in THF), 0 °C, 30 min, then 0.5 M HCl; (iii): for **134d**, X = Br: **134d** (1.0 equiv), *n*BuLi (1.05 equiv in THF), anh THF (9.5 mL) at −78 °C, 45 min; (*i*PrO)_3_B (2.5 equiv), −78 °C to rt, 2h, then Et_2_O (9 mL) and 1N HCl (9 mL) at 0 °C.

## Data Availability

No new data were created or analyzed in this study.

## Abbreviations

The following abbreviations are used in this manuscript:

Ac	Acetyl group
ACN	Acetonitrile
Ac_2_O	Acetic anhydride
AcOEt	Ethyl acetate
AcOH	Acetic acid
AD	Alzheimer’s disease
AIBN	Azobisisobutyronitrile
aq	Aqueous
AtOH	1-Hydroxy-7-azabenzotriazole
[BMIM][Cl]	1-Butyl-3-methylimidazolium chloride
[BMIM][Tf_2_N]	1-Butyl-3-methylimidazolium bis(trifluoromethylsulfonyl)imide
[BMIM][TfO]	1-Butyl-3-methylimidazolium trifluoromethanesulfonate
BTA	Benzothioxanthene anhydride
BTI	Benzoxanthene imide
Bu_3_SnH	Tributyltin hydride
Cbz	Benzyloxycarbonyl
CD_3_CN	Trideuteroacetonitrile
Cdk4	Cyclin-dependent kinase 4
(CD_3_)_2_SO	Deuterated dimethylsulfoxide
CNS	Central nervous system
conc	Concentrated
DavePhos	2-(Dicyclohexylphosphino)-2′-(dimethylamino)biphenyl
DCM	Dichloromethane
DFT	Density functional theory
DMA	*N,N*-Dimethylacetamide
DMF	*N,N*-Dimethylformamide
DMSO	Dimethyl sulfoxide
DSC	Differential scanning calorimetry
EDC·HCl	1-Ethyl-3-(3-dimethylaminopropyl)carbodiimide hydrochloride
EtOH	Ethanol
HL-60 cell	Human Leukemia-60 cell
IC_50_	Half-maximal inhibitory concentration
ILs	Ionic liquids
*i*Pr	Isopropyl
LCs	Liquid crystals
LEC	Liquid-encapsulated Czochralski
LEDs	Light-emitting diodes
MeI	Methyl iodide
MeOH	Methanol
MIC	Minimum inhibitory concentration
min	Minutes
MTBE	Methyl *tert-*butyl ether
MTT	(3-(4,5-Dimethylthiazol-2-yl)-2,5-diphenyltetrazolium bromide)
MWI	Microwave irradiation
OLED	Organic light-emitting diode
ORTEP	Oak ridge thermal ellipsoid plot
PAHs	Polyaromatic hydrocarbons
PDT	Photodynamic therapy
PS	(Proton Sponge): 1,8-bis(*N*,*N*-dimethylamino)naphthalene
psi	Pound square inch
PXRD	Powder X-ray diffraction
SAR	Structure–activity relationship
S_E_Ar	Electrophilic aromatic substitution
S_N_2	Substitution nucleophilic bimolecular
S_N_Ar	Nucleophilic aromatic substitution
SPhos	2-Dicyclohexylphosphino-2′,6′-dimethoxybiphenyl
TBHP	*tert*-Butyl hydroperoxide
*t*Bu	*tert*-Butyl group
*t*BuOH	*tert*-Butanol
*t*BuONO	*tert*-Butyl nitrite
TCI	Thiochromenocarbazole imide
TEA	Triethylamine
TEMPO	2,2,6,6-Tetramethylpiperidin-1-oxyl
terpy	Terpyridine
TFA	Trifluoroacetic acid
THF	Tetrahydrofuran
TMS_3_SiH	Tris(trimethylsilyl)silane
*t**rig*	Trigonal
TSI	Thiochromenothioxanthene imide
